# Summary of Social and Scientific Progress in America

**Published:** 1876

**Authors:** 


### SUMMARY OF SOCIAL AND SCIENTIFIC PROGRESS IN AMERICA.

The following summary of the social and scientific progress in America during the past hundred years, we copy from *Chicago Medical Journal:*

During the century we have gained possession of, and brought largely under subjection, the vast domain on which we dwell, with its wealth of fertility and rich deposits of minerals; established a government that is a model and example for nations struggling to be free· produced a galaxy of statesmen, generals, orators, philosophers, poets and scientists, who rival the fame of the classic Greeks and Romans, and whose names will lose their lustre only in the dissolution of the English language.

Two of the greatest inventions of modern times are of American origin: the steamboat, which carries trade and civilization to every quarter of the globe, and approximates the most distant countries; and the telegraph, that swift bearer of thought, which, even more than the steamboat, makes other lands seem near. The material benefit, moral improvement and great enjoyment afforded the human race by these two inventions, are entirely beyond our comprehension.

In agriculture we have taught mankind how to sow, cultivate and harvest by machinery, thus making manifold the capacity of every man who cultivates the soil.

Our nation has furnished many ingenious instruments and devices to lessen the labor of women, and now, in addition to the machinery for spinning, weaving and knitting, we have added the universally used sewing machine. And so we might continue on these general subjects until a large book was filled, but we refrain, and turn to a rapid enumeration and brief mention of some of the most important achievements in medicine.

Before proceeding, we stop to say that we are proud of our dentists, who are part and parcel of our great profession. American dentists, even before the Franco-German war, were the leading dental surgeons of the great cities of the continent. They were sought by the aristocracy of Paris, Berlin, Vienna, Florence, St. Petersburg, etc., as superior practitioners, and we are not informed that any change has taken place since that great struggle.

In Materia Medica we have made a number of discoveries worth special mention, among which is veratrum viride. The discovery of the peculiar properties of this plant and its therapeutical application, were made by an American practitioner. Its effects in diminishing the rapidity of the circulation are very remarkable, and form a basis upon which rests its claims as a remedy in inflammation ; and from much observation we are prepared to say that in pneumonia, pleurisy, cerebral inflammation, metro-peritonitis, and many other forms of inflammation, veratrumviride is a remedy of very great value. We merely mention a few others, as cimicifuga, eupatorium, gelseminum, lobelia, podophyllin, prunus virginiana, spigelia, senega, serpentaria, and sanguinaria.

Surgery has been enriched by the researches of American surgeons to a degree that does us great credit.

The war of the rebellion gave the members of our profession a field of vast opportunities, which was cultivated with an assiduity, intelligence and ardor that have resulted in many very important improvements in operations already established, and the introduction of new and valuable processes in surgery.

The profession in Europe have acknowledged the great service the surgeons of the armies on both sides of that great conflict have rendered to the practice of surgery.

Among other things, they have contributed largely to a correct knowledge of the subject of resection of the joints, the treatment of wounds in the chest, the comparative superiority of out-door or tent accommodations for the wounded, to the confinements of such patients in hospital, and the preference of coffee to alcohol as a stimulant for soldiers in the field.

In civil surgery a number of important operations have been given to the profession by American surgeons. The late Dr. Mott was the first to ligate the arteria innominata. Dr. Brainard instituted the valuable process of drilling the broken ends of the long bones to promote their uniou in ununited fractures, with variations to suit the taste of the operator. This has been adopted as a permanent improvement in that embarrassing condition of things.

The reduction of dislocated hip joint by manipulation, is now well understood and practiced as one of the great surgical improvements of our time.

Americans have done much to perfect and establish the operation of excision for caries of the bones related to the hip joint, to the systematic development of the plan of treating inflamed joints by extension, and the use of adhesive strips for extension in fractures.

It is now everywhere conceded that ovariotomy had its origin in America, and nobody seriously competes with our late great Kentucky surgeon, Ephraim McDowell, for the honor of having planned and executed this magnificent operation. He not only did the first successful ovariotomy, but he repeated, studied and worked it out in all its details, and taught it to the profession.

It is now universally acknowledged as a legitimate operation, practiced in all civilized countries, and by common consent its introduction is att.ibu.ed to its rightful and successful originator, and thus in doing tribute to its author, the surgeons of the world unite in according the highest honors to his country.

This great achievement has hardly a parallel in surgical enterprise, and the completeness of the operation, as it came from the hands of our renowned countryman, will appear the more remarkable when we consider the fact that some of the most successful operators now believe there has been little, if any, improvement upon his simple but scientific method of performing it, notwithstanding the many ingenious devices added by his successors.

Second only in importance to the original operation, is the practical and philosophical method of enucleating the tumor—taught by an eminent American cotemporary—under circumstances that render its removal otherwise almost impracticable.

The brilliant operation of removing an ovarian tumor by vaginal incision, may be ennumerated among the recent triumphs of American surgery, which must eventually take rank as a practical and legitimate proceeding, under circumstances that will be prescribed by future observation. Vaginal ovariotomy for the induction of the menopause in certain grave forms of chronic ovarian and uterine affections, not amenable to other methods of treatment, is now on trial in this country.

If our memory is correct, the method of rapid reduction of chronic inversion of the uterus, has been perfected, if it did not originate, in this country, and one of our eminent gynecologists has had better success in the practice of it than has hitherto been attained. The enucleation of intramural fibrous tumors of the uterus, is another operation almost wholly American, and the gynecologists of this country were the earliest to practice it extensively and successfully. The indications justifying a resort to it, the instruments with which to perform it, and all the manipulation and different steps of the operation, have been so minutely and lucidly described and illustrated by two living gynecological surgeons, that it has been divested of many of its difficulties, and become practicable by practitioners of ordinary skill and experience.

The world is also indebted to American surgery for the only uniformly successful method of curing vesico-vaginal fistula. Although operations of various plans had been devised by many of the gifted surgeons of Europe, it remained for an American to simplify and bring it within the capacity of the general surgeon.

We think it is not too much to say that its successful per formance was barely possible in the hands of the most experienced surgeons, while now we hear of its being practiced all over the country, and it is no longer regarded as an operation of extraordinary difficulty. The numerous difficulties, previously insurmountable, have been met and overcome by the imdomitable persevereance of our professional compeers in this country.

Growing out of the experiences in that operation is the introduction of silver wire for sutures in certain plastic operations, and now it is used extensively here and abroad.

But the crowning event of the century, if not of all past time, is the American discovery of the means and methods of producing anæsthesia.

There is no discovery in the annals of medicine comparable to it except that of vaccination. It has created an era in the practice of surgery and obstetrics, and added an important therapeutical process in the general practice of medicine. The glory of this discovery is not shared by any but Americans, and it is an additional gratification to us to know that surgical authorities, the world over, are returning to *our anæsthetic* as the most safe and reliable.

As now manufactured by several of our pharmaceutical chemists, ether is very nearly as speedy in its effects, and quite as effectual in causing a profound influence, as chloroform, or any of the newer compounds so highly praised.

With a record, feebly indicated by this very imperfect summary, as the result of the first hundred years of our national life, we are justified in expecting for the next century, such achievements as will make us rank with the most advanced of nations in every respect.

